# Effects of Climate Change and Drought Tolerance on Maize Growth

**DOI:** 10.3390/plants12203548

**Published:** 2023-10-12

**Authors:** Kyung-Hee Kim, Byung-Moo Lee

**Affiliations:** Department of Life Science, Dongguk University—Seoul, Seoul 04620, Republic of Korea; redanan@dongguk.edu

**Keywords:** drought stress, heat stress, molecular breeding, quantitative trait locus (QTL), maize yield

## Abstract

Climate change is affecting all regions of the world with different climates, and the scale of damage is increasing due to the occurrence of various natural disasters. In particular, maize production is highly affected by abnormal climate events such as heat waves and droughts. Increasing temperatures can accelerate growth and shorten the growing season, potentially reducing productivity. Additionally, enhanced temperatures during the ripening period can accelerate the process, reducing crop yields. In addition, drought stress due to water deficit can greatly affect seedling formation, early plant growth, photosynthesis, reproductive growth, and yield, so proper water management is critical to maize growth. Maize, in particular, is tall and broad-leaved, so extreme drought stress at planting can cause leaves to curl and stunt growth. It is important to understand that severe drought can have a detrimental effect on the growth and reproduction of maize. In addition, high temperatures caused by drought stress can inhibit the induction of flowering in male flowers and cause factors that interfere with pollen development. It is therefore important to increase the productivity of all food crops, including maize, while maintaining them in the face of persistent drought caused by climate change. This requires a strategy to develop genetically modified crops and drought-tolerant maize that can effectively respond to climate change. The aim of this paper is to investigate the effects of climate change and drought tolerance on maize growth. We also reviewed molecular breeding techniques to develop drought-tolerant maize varieties in response to climate change.

## 1. Introduction

**Figure 1:**
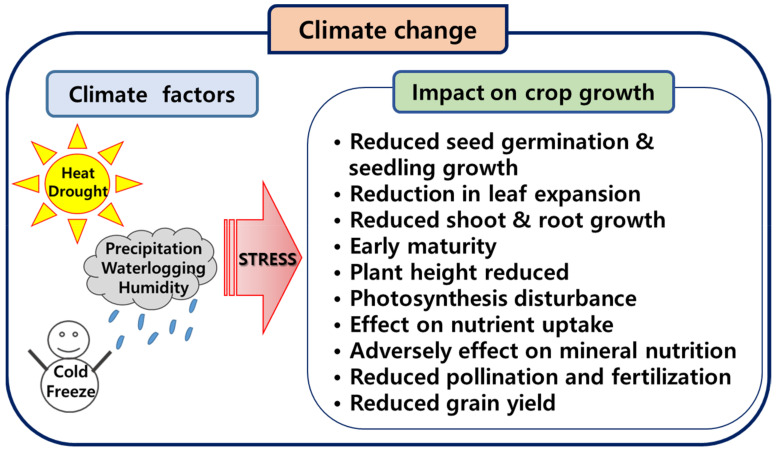
Effects of climate change on crops.

Global warming is caused by human activity, primarily through greenhouse gas emissions, and between 1850 and 1900, and 2011 and 2020, the Earth’s surface temperature increased by 1.1 °C [1]. Climate change caused by humans is already one of the main contributors to climate change in all regions of the world. This has resulted in widespread negative impacts, including loss of and damage to nature and people. The relationship between population growth and climate change is also becoming an important issue as it affects agricultural production. The impact of climate change on crop production varies by region and crop, and climatic factors such as temperature, precipitation, and humidity also affect crop development (Figure 1). Crops are susceptible to abiotic stresses, such as drought and temperature extremes, which can result in growth inhibition [2]. The major features of these stresses can reduce crop vigor, inhibit growth and development, and reduce crop yield. In particular, extreme weather events such as heat waves and droughts are important factors that severely affect maize production. These factors cause drought damage, inhibit maize growth, and significantly reduce yield [3].

In addition, climate change is making droughts and heat waves more severe and prolonged, and water supplies more difficult to obtain. This makes it more difficult for crops to survive in extreme conditions caused by water scarcity. Maize is generally tall and broad-leaved, and a severe drought during the seedling or growing period causes leaf curling and stunted growth [4,5,6,7]. Water scarcity before and after flowering also affects maize yield. Therefore, adequate water supply is required during this period. Maize is subjected to drought stress due to a lack of moisture, which affects seedling emergence, early vegetative growth, photosynthetic capacity, reproductive growth, fertilization, seed formation, and yield [3,8,9]. Drought stress inhibits pollen production by interfering with the induction of anthesis in male flowers [10]. In addition, drought stress during flowering may have a greater effect on the development of female flowers (silks) than male flowers, significantly reducing kernel set.

**Figure 2:**
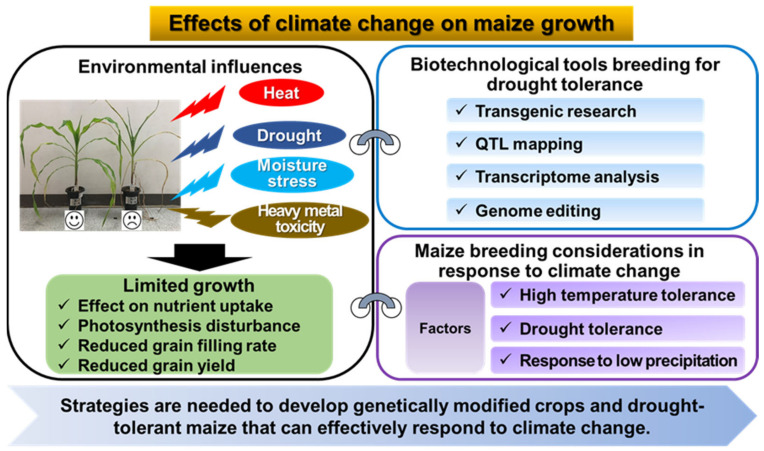
Maize breeding strategies to respond to climate change.

Meanwhile, climate change not only causes drought stress, but can also acidify soils and lead to pollution such as heavy metals. Heavy metal toxicity in soils is a major environmental and ecological concern, mainly due to the increasing heavy metal contamination of agricultural land and soils, leading to the introduction of heavy metals into crops [11,12,13]. Non-essential heavy metals, even in trace amounts, are toxic to plants and affect crop growth [14]. Cadmium (Cd), in particular, is relatively more soluble than other trace elements in the soil, so it easily enters the natural environment and accumulates in the roots of plants [15]. In order to preserve and restore the ecological functions of these soils, it is necessary to remediate Cd-contaminated soils. A recent study used maize to screen for Cd-tolerant varieties and to investigate the variety responses to the induction of Cd toxicity [16]. Increased Cd levels reduced maize growth and biochemical traits, and cadmium generally accumulated maximally in roots rather than leaves. In addition, some maize cultivars showed superior growth and tolerance to Cd stress. Some maize genotypes are tolerant to Cd-contaminated soils [17], making maize a potential crop for phytoremediation, especially in Cd-contaminated soils [18,19]. Currently, maize production is declining due to the accumulation of heavy metals in the soil. Finding resistant maize varieties is necessary to meet the food needs of the world’s growing population. This review examines the effects of climate change and drought tolerance on maize growth and reviews molecular breeding techniques for developing drought-tolerant maize varieties in response to climate change (Figure 2). We have also organized our findings into sections that provide an update on the physiological responses of maize to stress and biotechnological tools for breeding drought-tolerant maize. Finally, we have summarized environmental factors and scientific techniques as key factors to consider when breeding maize for climate change. We hope that this review can provide researchers with a wealth of knowledge on climate change and maize growth response.

## 2. Effects of Climate Change on Maize Growth

Climate change could have a serious impact on maize growth and production. Maize is a staple crop in many parts of the world and plays an important role in global food security. The impact of climate change on maize may vary depending on local climate patterns, but some common impacts include the following factors.

### 2.1. Drought Stress

Droughts are expected to become more frequent as temperatures rise and rainfall deficits increase. Drought stress during the reproductive or growth stage has the most devastating effect on crop yields. Drought severely affects agricultural production by affecting plant growth, reproduction, and physiology [20]. Field experiment data published between 1980 and 2015 showed that drought (approximately 40% water loss) reduced wheat and maize yields by 20.6% and 39.3%, respectively [21]. Maize was more sensitive to drought than wheat, especially during the reproductive stage, and was sensitive in both arid and non-arid regions. In maize, hot and dry weather during pollination and fertilization can have a negative effect on yield. In particular, drought stress can reduce overall plant size, ear size, and kernel number. Drought stress typically affects final yield during the vegetative growth stage of maize (up to V12) [22]. Maize is generally most susceptible to stress during pollination and fertilization; in particular, if drought stress continues for two weeks before pollination and the plant wilts, yields can be reduced by up to 3~4% per day [23]. Depending on the level of stress during silk and pollen development, yield losses can be up to 8% per day. In addition, if drought stress persists for two weeks after silking occurs, yield losses can reach up to 6% per day.

In maize, pollination is the process of transferring pollen from tassels to ears. When silk pollen germinates, it forms a pollen tube that delivers genetic material to each ovule in the ear, and the fertilized ovule becomes a maize kernel. However, severe drought and heat stress can disrupt the synchrony between pollen availability and silk emergence [24]. It can also dry out exposed silk, making it unable to accommodate captured pollen grains. Meanwhile, high-temperature damage during the pollination process in maize most often occurs together with drought stress, but it can also occur alone. In most cases, low relative humidity and temperatures above 35 °C dry exposed silk but have little effect on silk elongation. Pollen, on the other hand, is more likely to be damaged or die at temperatures above 32 °C and low relative humidity. Studies have shown that prolonged temperatures above 32 °C can have a negative effect on moisture and grain filling [25]. Fortunately, pollen release typically occurs from early to mid-morning, so that over time, fresh pollen is available every morning until pollen maturation occurs and pollen production is complete.

### 2.2. Heat Stress

All plants have an optimal temperature range for growth, and temperatures outside this range have negative effects on growth and development. The gradual increase in annual mean temperature and heat is one of the most threatening abiotic stresses. Ultimately, rising temperatures can alter crop development and growth, affecting the yield and quality of agricultural products [26]. Higher temperatures may benefit some crops in cooler regions, but in most cases, higher temperatures have a negative effect on crop productivity and reduce yields. The Crop-Environment Resource Synthesis (CERES) Maize model was first used to simulate historical maize yield variability in nearly 200 regions of sub-Saharan Africa [27,28]. The study simulated a hypothetical future scenario in which a 2 °C increase in temperature would cause a greater reduction in maize yields in sub-Saharan Africa than a 20% decrease in precipitation. The temperature threshold for damage from heat stress in reproductive organs is significantly lower than in other organs [29]. For maize to form a kernel, it requires the production of viable pollen, its transfer to the germ cell of the male gamete, and the initiation and maintenance of embryo and endosperm development [30]. High temperatures, particularly during reproductive growth, are associated with reduced yield due to reduced grain number and weight. In general, fertilization at high temperatures can reduce the number of ovules that develop into kernels. In addition, the tassels, the reproductive organs for pollen production, are sensitive to heat stress, affecting their morphology and physiological function [30]. In particular, the outer membrane of the anther is so thin that high temperatures are easily transmitted to the anther, causing deformation and physiological damage [31,32]. Heat stress, especially at the six visible leaf collar (V6) stage, can lead to the formation of underdeveloped tassels, eventually leading to tassel blast (TSBL) and complete desiccation. Furthermore, reproductive tissue damage in the form of TSBL reduced the number of viable pollen [33]. In general, maize exposed to heat stress from the ninth leaf stage (V9) to the tasseling stage (VT) slows tassel growth, destroys anther structure and reduces pollen viability, resulting in a shorter pollination period [34,35].

### 2.3. Moisture Stress and Precipitation Variability

Climate change may alter precipitation patterns, causing more frequent and severe droughts in some regions and increased precipitation in others. Droughts are increasing globally due to decreased rainfall and changing rainfall patterns. Precipitation is the meteorological factor that has the greatest impact on maize productivity. The flowering period is the most susceptible to drought damage, and yields decrease depending on the time and extent of damage [36]. During the week following silk development, water deficit significantly reduces kernel weight and maize yield continues to decline by approximately 30%. In addition, the tasseling stage is a drought-sensitive period [37], and when moisture stress occurs before tasseling, the occurrence of tasseling and silking is delayed by more than 2 weeks and the yield is reduced by more than 90% [38]. In another study, the anthesis–silking interval (ASI) increased by 2–3 days when moisture stress occurred in the early stages of tasseling, and the ASI increased by 0.75–1.25 days when water was deficient during the anthesis period [39]. Therefore, water deficit increases ASI but reduces ear length, silk growth, seed weight, and yield. Additionally, when water stress occurs during fertilization, pollen release and silk development are inhibited, resulting in a 3–8% reduction in maize yield [40]. If moisture stress occurs during the grain filling stage, the daily yield reduction rate is 3–5.8%, and if extreme moisture stress occurs during this period, maize yield may decrease by 20–30%.

Meanwhile, the frequency of soil moisture stress is increasing due to climate change, reducing the stability of field crop cultivation. According to research results on the effects of soil moisture management methods at each growth stage of maize on growth and yield, intensive soil moisture management was required before V8 (vegetative stage; eighth leaf), and 100–500 kPa (20.3–27.8%) management was required from V8 to VT (tasseling stage; lowest branch of the tassel is visible) [41]. No additional irrigation was required after VT. After the flowering period, there was no significant reduction in growth and yield even without separate irrigation in the later stages of growth. In the later growth stages, irrigation efficiency can be increased, and yield losses minimized without additional irrigation. In maize, moisture stress, such as drought between V8 and VT, is directly related to yield, including ear formation and maturity, and depending on the degree of stress, yield can be decreased by up to 50% [38,40].

## 3. Effects of Drought and Heat Stress on Maize Yields

**Figure 3:**
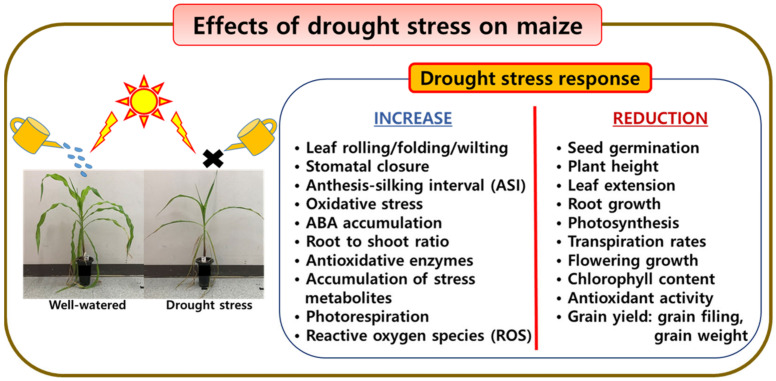
Effect of drought stress on maize growth and development.

Drought stress is an abiotic factor that affects maize growth and yield [3,20,42,43,44,45] (Figure 3). In general, seed yield and harvest index decrease as the severity of drought stress increases.

Climate change caused by increased greenhouse gas emissions threatens crop yields and food security by increasing temperatures and changing precipitation patterns [46]. As temperatures rise, drought occurs due to the rapid loss of moisture from plant tissues and soil surfaces, and excessive temperatures can directly damage crops [47]. Drought and heat stress, alone or in combination, have severe impacts on agriculture and are critical to food security. At critical stages of maize growth and development, prolonged drought and extreme climate variability significantly reduce crop yields and crop losses [48]. Irregular rainfall fluctuations have led to a steady decline in maize production, and maize area has also decreased to adapt to periods of drought. In addition, abiotic stresses such as drought and heat that occur simultaneously during the crop growth stage can have a serious impact on crop production. These abiotic stresses are known to influence the emergence and spread of pathogens, insects, and weeds, and can also lead to potentially dangerous pests [49]. Drought affects crop growth and yield by affecting photosynthesis [50], causing wilting, slowed growth, delayed leaf emergence, and reduced leaf area, especially at the seedling stage [51]. In maize, the release of pollen and the development of maize silk during the flowering period are closely related, so yield is determined at this time, and there is also a close relationship between the final yield and the ASI. Therefore, drought stress during flowering delays the development of maize silk and reduces yield [52,53].

In particular, the two most important factors affecting corn production are drought and heat. There are several studies on the importance of these factors for maize yield in different parts of the world [54]. In the Czech Republic, total maize yields increased from 1961 to 2010, but yields decreased after 2010 when precipitation decreased [55]. This is because rising temperatures since 2010 appear to be correlated with overall production and production decline. In Khyber Pakhtunkhwa (Pakistan), rainfall had a positive effect on maize production from 1996 to 2015, while temperature increases had a negative effect [56]. Climate change will reduce maize yields in Europe by 20% by 2050 [57]. Drought and heat stress are also major causes of yield loss. Turkey also expects maize yields to decline by 10.1% by mid-century due to drought and heat stress [58]. In the United States, projections of changes in precipitation and temperature suggest that maize yields will decline by 39 to 68 percent by 2050 compared to the 2013–2017 period, depending on the climate scenario [59].

## 4. Application of Biotechnological Tools Breeding for Drought Tolerance in Maize

Drought and heat stress are reducing the yields of major food crops and increasing risks to global food security [60,61]. The development of drought-tolerant lines in maize is becoming increasingly important. Drought tolerance is the ability of plants to sense water scarcity and activate appropriate response pathways to maintain viability and reproductive capacity. Among drought tolerance mechanisms, drought escape strategies typically involve ensuring the successful completion of the life cycle by reducing the time required for plants to transition to reproductive developmental stages or promoting entry into a dormant state until environmental stressors are removed [62,63]. Another mechanism of drought resistance is drought tolerance. Drought tolerance occurs through the activation of stress response mechanisms under water limitation through the biosynthesis of osmotically protective small molecules, the increased production of antioxidant enzymes, and the switching of phytohormone regulatory pathways [64,65].

Crops are often exposed to drought stress during the early stages of growth, when germination and seedling growth are hindered by a lack of water [66,67,68]. In addition, water management is important because vegetative and reproductive growth after initial growth is sensitive to drought stress. Drought stress affects maize production, particularly during flowering and grain filling. Increasing drought resistance is therefore a major goal of maize breeding to prepare for climate change. Traditional plant breeding develops genetically improved crops by crossing superior plants with different genotypes and then selecting the progeny through years of testing for improved yield performance under drought stress [69]. However, this process is time-consuming and requires patience as it is repeated with each generation and continues for many years. Conventional breeding methods have been used to develop new maize varieties, but these methods do not guarantee improved yield and stress tolerance [70]. Therefore, new techniques are needed to improve selection efficiency. To complement this, biotechnological approaches, including genetic modification, genome-wide marker-assisted selection, transcriptome analysis, and gene editing technologies, have recently provided more direct, efficient, and accurate approaches to trait improvement [71,72,73].

### 4.1. Transgenic Research to Develop Drought-Tolerant Maize

Plant transformation studies are useful for functional analysis of genes involved in various stress responses and adaptations and can be used to transfer useful genes to other crops [74]. Transgenic maize with increased *ZmNF-YB2* expression was shown to be drought tolerant and to maintain photosynthetic capacity, and improvements in grain yield were observed over several growing seasons in water-stressed fields [75]. In addition, transgenic maize with increased *ZmNF-YB2* expression exhibits drought tolerance in response to several stress-related parameters, including chlorophyll content, stomatal conductance, leaf temperature, and reduced wilting. The transgenic drought-tolerant maize line *SbSNAC1-382* (overexpression of the *SbSNAC1* gene), developed using the single-molecule real-time (SMRT) sequencing, showed improved tolerance to drought stress [76]. In addition to these mentioned transformation studies, *Agrobacterium*-mediated transformation methods are helpful in improving drought tolerance and yield in maize. Table 1 describes transformation studies related to *Agrobacterium*-mediated drought tolerance.

To identify the genetic components underlying drought tolerance in maize, maize drought tolerance at the seedling stage has been analyzed using genome-wide association studies (GWAS) [81]. Studies have identified natural variation in *ZmVPP1* as the most major contributor to drought resistance, and drought-inducible expression of *ZmVPP1* conferred drought-tolerant genotypes. In addition, transgenic maize with enhanced *ZmVPP1* expression showed improved drought tolerance with enhanced photosynthetic efficiency and root development. *ZmASR1* is one of the most highly expressed abscisic acid-, stress-, and ripening-induced (*ASR*) proteins in maize [82]. Overexpression of *ZmASR1* increased leaf dry weight and total chlorophyll content under water-limited conditions and had a significant effect on improving maize yield. The *ZmSOPro* gene was isolated from the maize genome and analyzed for its role in drought tolerance. Minimal *ZmSOPro* was significantly activated by ABA or drought stress in transgenic maize plants. This *ZmSO* gene and its characteristic 119 bp promoter are potential candidates for genetic engineering of drought tolerance in maize [83]. In maize, gibberellin (GA) content is correlated with drought tolerance, but the molecular mechanism is still unclear. *AtG2ox1*, a member of the *GA2ox* family with well-defined functions, was used to generate GA-deficient transgenic maize. As a result, overexpression of *AtGA2ox1* regulated GA levels and improved the drought tolerance of transgenic maize [84].

### 4.2. QTL Mapping for Drought Tolerance in Maize

Quantitative trait loci (QTL) mapping analyzes the genetic basis of complex traits and is used as a basis for marker-assisted selection (MAS). QTL mapping can determine whether a chromosomal fragment between two specific breakpoints is associated with a particular phenotype. Several studies have performed QTL mapping for drought tolerance in maize (Table 2). QTL analysis associated with flowering time and ASI in maize was investigated under well-watered conditions and two water stresses (moderate and severe stress) [53]. For ASI, six QTL were identified on chromosomes 1, 2, 5, 6, 8, and 10 under drought conditions. Under water stress, four QTL were common to the expression of male flowering (MFLW) and female flowering (FFLW), one QTL was common to the expression of ASI and MFLW, and four QTL were common to the expression of ASI and FFLW. A recent study conducted a meta-QTL analysis by summarizing 542 QTL (238 for drought, 61 for flood, 82 for heat, 93 for cold, and 68 for salinity) reported in 33 abiotic stress tolerance papers [85]. Among these, 32 meta-QTL with a total of 1907 candidate genes were detected against different abiotic stresses were detected in different genetic and environmental backgrounds.

Meta-QTL analysis of reported QTL can identify stable/true QTL, and these QTL can facilitate drought tolerance breeding in maize. Another meta-QTL study identified mQTL for grain yield (GY) and anthesis–silking interval (ASI) for 18 parental maize populations evaluated under identical conditions in managed water stress and well-watered environments [98]. The meta-analysis identified 68 mQTL (9 QTL specific for ASI, 15 specific for GY, and 44 for both GY and ASI). In another study, 54 QTL were reported to influence different photosynthetic traits in maize under well-watered and drought stress conditions [99]. A total of 43 QTL identified under drought stress indicate that tolerance to photoinhibition is a key factor influencing drought tolerance in maize. These results provide insight into the genetic mechanisms responsible for photosynthesis under different water availability conditions and can potentially be used for the MAS-based development of drought-tolerant maize varieties [99]. QTL sequencing analysis is one of the most reliable methods for identifying potential QTL/genes underlying drought tolerance in maize. Studies using this method have used leaf relative water content (LRWC) as a drought tolerance index to evaluate drought tolerance in maize [100]. Through QTL-seq analysis, four QTL, qLRWC2, qLRWC10a, qLRWC10b, and qLRWC10c, were identified for LRWC under water deficit conditions. These QTLs will help to elucidate the molecular basis of drought tolerance in maize seedlings and may be useful for future functional analysis and related studies in the breeding of drought-tolerant maize varieties. Meanwhile, it has been confirmed that water stress affects leaf temperature in maize [88]. Stomatal responses to drought were observed in leaves, confirming physiological evidence that leaf temperature responds to drought. Additionally, to identify QTL, the research team established a mapping population of 187 recombinant inbred lines (RILs) derived by crossing Zong3 with 87-1 and used it to detect nine QTL associated with drought and leaf temperature.

Strategies for using MAS in drought tolerance QTL studies and breeding programs in maize and other crops have been extensively discussed in several reviews [101,102,103,104]. Previous QTL studies in maize have not identified QTL with effects large enough to be used effectively in MAS programs [105]. Following the analysis of maize QTL for yield-related traits in 1987, numerous maize researchers around the world began to develop molecular markers to tag genes/QTL for various traits of agricultural and scientific interest [106]. Molecular markers were used to identify QTL associated with various traits such as yield, purity, environmental adaptation, pest resistance, maturity and heat stress tolerance. Since the first genetic analysis of drought tolerance using QTL analysis was performed in maize in 1995 [107], many studies have been conducted to identify QTL that the control yield or important ecological and physiological traits under limited water supply in maize. Many papers and studies have been devoted to the study of QTL for drought tolerance in maize and the strategies promoted to apply QTL for MAS in breeding programs [101,102]. Genetic analysis of maize development in drought-prone environments can greatly benefit from the use of DNA markers [108,109], but to date, few have been directly applied to actual maize breeding programs. Because QTLs are specific to the original genetic background of each QTL and the influence of a single gene for the trait of interest, the ability to improve drought tolerance in breeding trials using a large number of markers is limited. This is due to the complexity of the genetic basis and the influence of the genetic background, with QTLs being influenced not only by the developmental stage and environment of the plant, but also by the time and cost of accurately mapping maladaptive phenotypes, and by intergenic effects [105,110,111].

### 4.3. Impact of Transcriptome Analysis for Drought Tolerance in Maize

To analyze developmental and drought stress-related splicing changes in maize, thousands can be identified through the deep sequencing of leaves, ears, and tassels, as well as publicly available data on seed, endosperm, and embryo development [112]. Transcriptome analysis is widely used to identify gene expression for stress response and adaptation, to facilitate genetic dissection, and to study gene expression regulation in response to drought stress in maize [112,113]. To evaluate the impact of drought stress on developmentally regulated genes, a total of 94 RNA-seq libraries were collected from maize ears, tassels, and leaves of the open inbred maize line B73 at four developmental stages under well-watered and water-stressed conditions [112]. These results show that alternative splicing is closely related to tissue type, developmental stage, and stress conditions. Maize yield is determined by the successful development of the female inflorescence, the ear. In a related study, the inbred maize line B73 was examined at different developmental stages to investigate its response to drought stress [113]. It was found that drought inhibited plant growth but had little effect on the progression of developmental stages. These results demonstrate tissue-specific differences in response to drought stress through the parallel RNA-seq profiling of leaves, ears, and tassels at different developmental stages. In drought-stressed ears, genes regulating DNA replication, cell cycle, and cell division were significantly downregulated, consistent with the inhibition of ear growth under drought conditions [113]. In addition, transcriptome-wide association study (TWAS) and mendelian randomization (MR) analyses were developed to reveal the association between drought tolerance and gene expression changes [114,115]. A study identified the genetic architecture of leaf cuticle conductance in maize using GWAS and TWAS analyses [114]. Of the 22 candidate genes identified, 4 were involved in the biosynthesis and export of cuticle precursors, 2 in cell wall modification, 9 in intracellular membrane transport, and 7 in the regulation of cuticle development. These results provide insight into the role of regulatory changes in the development of the maize leaf cuticle.

### 4.4. Genome Editing: Genetic Improvement of Drought Tolerance in Maize

Recently, genome editing techniques have been developed to rapidly and accurately manipulate DNA sequences by editing key genes to develop drought-tolerant germplasm. Clustered Regularly Interspaced Short Palindromic Repeats (CRISPR)-Cas9-based genome editing technology is being used to increase disease resistance in crops and improve tolerance to abiotic stresses such as drought and heat [116]. Currently, research on maize drought tolerance using genomic technology is diverse, and crop molecular breeding technology for crops is continuously being updated (Table 3). *ARGOS8*, which is involved in ethylene biosynthesis, has been used to develop drought-tolerant maize. A new strain of *ARGOS8* was created using CRISPR/Cas9 technology, and lines were identified that increased yield by up to five bushels per acre when *ARGOS8* maize was subjected to drought stress at flowering [117]. CRISPR/Cas9 technology can be used to generate new drought-resistant mutants. In general, cuticle wax is a natural barrier for plant organs that protects plants from damage caused by various stresses. This cuticle wax-related gene, *Zea mays* L. SEMI-ROLLED LEAF 5 (*ZmSRL5*), was generated as a biallelic mutant using CRISPR/Cas9 technology [118]. *ZmSRL5* encodes the CASPARIAN-STRIP-MEMBRANE-DOMAIN-LIKE (CASPL) protein, which is located in the plasma membrane and is highly expressed in developing leaves. They found that *ZmSRL5* is required for the structure, and that proper maintenance of the cuticle wax structure in maize may increase drought resistance. In another study, CRISPR/Cas9 technology was used to generate *zmslac1-2* and *zmcpk37* mutants. Transgenic maize lines overexpressing *ZmCPK35* and *ZmCPK37* showed improved tolerance and increased yield under drought conditions. *ZmSLAC1* plays an important role in stomatal closure in maize, which is regulated by *ZmCPK35* and *ZmCPK37* [119]. In addition, the increased expression of *ZmCPK35* and *ZmCPK37* can improve maize drought tolerance and reduce yield loss under drought stress. In maize, the expression of *abh2* gene was found to be negatively associated with drought tolerance in plants. To identify this gene, we used CRISPR/Cas9 technology to knock out the *abh2* gene and generate *abh2*-CRISPR in maize (three independent homozygous lines: *i1*, *d2*, and *d35*) [120]. All three mutants had higher survival rates under drought than wild-type plants. Additionally, it was confirmed that stomata close faster in response to water deficit stress and are resistant to drought stress. Crop production methods using gene knockout editing can improve stress tolerance by directly targeting genes whose gene expression is negatively associated with stress tolerance. The use of CRISPR/Cas9 in maize is limited for breeding for drought tolerance. The use and efficiency of CRISPR/Cas9 can be extended by editing target genes to generate desired mutations in maize.

## 5. Future Prospects of Maize Breeding Research in Response to Climate Change

Climate change due to continued global warming leads to geographical and seasonal changes, resulting in changes in the agro-climatic zone. As a result, agricultural production is indirectly affected, including changes in crop adaptation zones, fertility, weeds, and pests. Increasing atmospheric carbon dioxide concentrations and rising temperatures can affect photosynthesis in crops, reducing growth rates and water use efficiency. This has a direct impact on crop productivity. Maize also loses around 15% of its global yield due to drought stress. Therefore, to prevent these yield losses and to maintain maize yields in dry regions, the drought tolerance of maize must be improved [123]. Through numerous studies, researchers have uncovered the genetic structure and regulatory mechanisms involved in maize drought tolerance. In particular, drought-related QTL analysis has identified genomes that can select for specific alleles. GWAS has also been used to identify many genetic variants associated with drought in maize. However, most of the candidate genes identified through GWAS are unidentified genes and require additional genetic analysis. To complement this, we are linking multi-omics research on droughts and analyzing linkage mapping together. Linkage analysis expands the scope of drought-related genetic information and uses it effectively. Recently, proteomics analysis has been used in maize breeding to provide functional information for interpreting gene expression variation [124]. Compared to the transcriptome, subtypes classified by the proteome show higher accuracy, similar to genomic subpopulations. Understanding the molecular regulatory mechanisms of drought responses using these analytical techniques can also provide useful information for modern maize breeding. It will also help to develop new maize varieties that are better able to adapt to the challenges of water scarcity. Maize breeding in response to climate change aims to develop maize varieties that can reduce negative impacts on agriculture such as high temperatures, droughts, and heat waves. The following table describes key factors to consider when breeding maize for climate change (Table 4).

There is an urgent need to develop maize varieties that are resistant to stresses such as drought and high temperatures, or that can adapt to changing ecosystems such as climate change. However, while the development of new maize varieties is important, the introduction of new cultivation technologies is also necessary. For example, it is important to develop agronomic technologies that can maintain productivity and quality by adjusting planting and transplanting times for maize in response to climate change. In addition, water use efficiency must be considered as the drying area increases. There is a need to develop technologies to capture water to conserve soil moisture or, conversely, to prevent nutrient leaching. In the future, climate change may be more rapid than today, so researchers must work to ensure that all crops can adapt to climate change.

## 6. Conclusions

Continued population growth and increased consumption increase greenhouse gas emissions through climate change. Rising temperatures and changing rainfall patterns also threaten crop yields and food security. Drought and heat stress, alone or in combination, have a major impact on agriculture and pose a risk to food security. Recent drought stress is reducing maize growth and yields over increasingly large areas and creating imbalances in food supply chains. In general, when maize is under drought stress, seedling emergence, vegetative growth, root development, photosynthesis, flowering, ASI, seed formation, and yield are severely affected. Researchers are developing new methods, including improving existing molecular breeding techniques and improving the genetic makeup of crops, to manage drought stress and develop drought-resistant varieties. Until recently, scientists have been working to develop a variety of molecular breeding techniques to combat the growing threat of climate change. In general, the main goal of maize breeding to prepare for climate change is to improve drought resistance. Therefore, researchers are using transformation studies, QTL mapping, transcriptome analysis, and genome editing to develop crops with genetically enhanced drought tolerance. Currently, various studies are underway to create a database using the whole genome of all crops and molecular marker technology for drought-resistance breeding. Meanwhile, ensuring yield stability requires extensive knowledge of the mechanisms responsible for yield loss and advances in molecular and phenotypic analysis tools. This may provide a basis for achieving productivity improvements within maize molecular breeding systems to respond to drought. However, no matter how advanced biotechnology and breeding techniques become, there are limits to manipulating nature. Currently, climate change is expected to have a negative impact on global food production. In developing countries, where 69% of the population is employed in agriculture, the impact of climate change on agriculture will not only affect food production, but also the entire national economy. Therefore, to mitigate the effects of climate change and ensure food security, multifaceted efforts are needed to adapt to climate change, including reducing greenhouse gas emissions and contributing to climate change mitigation through carbon sequestration. In conclusion, the development of molecular breeding technology to respond to climate change can provide excellent research results and can be applied as a new breeding technology in the development of new drought-tolerant maize varieties. To prepare for climate change, research is also needed to introduce cropping systems and develop cultivation techniques that allow crops to adapt to changing environments. Finally, molecular breeders will have to work harder to develop smarter, more focused, practical solutions to develop the technology to use molecular markers in breeding.

## Figures and Tables

**Table 1:** List of studies on drought tolerance of maize using transformation technology.

Target Gene	Technique	Comments	Reference
*ZmNF-YB16*	*Agrobacterium*-mediated method	*ZmNF-YB16* overexpression: maintains higher photosynthesis, improves drought stress resistance, and increases grain yield under drought stress conditions	[77]
*nced, rpk*	*Agrobacterium*-mediated method	Development of drought-resistant maize by introducing two genes involved in the ABA pathway and manipulating ABA signaling	[78]
*betA*	*Agrobacterium*-mediated method	Enhanced Glycine Betaine Accumulation: Improved osmotic/drought stress tolerance in transgenic maize	[79]
*ZmSDD1*	*Agrobacterium*-mediated method	Overexpression of *ZmSDD1*: reduced stomatal density and transpiration rate, improved drought tolerance	[80]

Abbreviations: ABA abscisic acid.

**Table 2:** List of QTL for drought tolerance traits in maize.

Population	Trait	QTL	Reference
Ac7643 x Ac7729/TZSRW RIL	Leaf elongation rate in correspondence with ASI	5	[86]
Ac7643 x Ac7729/TZSRW RIL	Seedling root traits in PEG solution	13	[87]
Ac7643S5 x Ac7729/TZSRWS_5_ F_2_ families	Flowering parameters, ASI	7	[53]
Zong3 x 87-1 RIL	LTD, RSDW, ESFW	9	[88]
Lo964 x Lo1016 NIL F_3:4_ families	Root trait and yield	1 (*root-yield-1.06*)	[89]
X178 x B73 F_2:3_ families	Yield and ASI	2	[90]
SD34 x SD35 F_3_ families	Yield, plant height, days to silking, ear number	5	[91]
CML444 x MALAWI RIL, CML440 x CML504 F_2:3_ families, CML444 x CML441 F_2:3_ families	GY and ASI	GY: 83, ASI: 62	[92]
Os420 (high L-ABA) x IABO78 (low L-ABA) F_3:4_ families	L-ABA	16	[93]
Os420 (high L-ABA) x IABO78 (low L-ABA) F_4_ families	L-ABA and yield	17	[94]
H082183 (drought-tolerant) x Lv28 (drought sensitive)	Yield	2 (*qEL4s, qKW4s*)	[95]
Huangzaosi x Mo17 RIL F_7_ families	Flowering time: DTA	2 (*qDTA3-3, qDTA10*)	[96]
Han21 (drought-tolerant) x Ye478 (drought sensitive) BC_3_F_6_ families	GY, ESP, ASI	2 (*qWS-GY3-1, qWS-ESP3-1*)	[97]

Abbreviations: ASI anthesis-silking interval, LTD leaf temperature differences, RSDW relative shoot dry weight, RSFW relative shoot fresh weight, GY grain yield, ABA abscisic acid, L-ABA leaf ABA concentration, DTA days to anthesis, ESP ear setting percentage.

**Table 3:** List of studies on drought tolerance of maize using genome editing technology.

Technique	Comments	Reference
CRISPR/Cas9 system to edit *ARGOS8* Delivery: particle bombardment	Flowering: Increases grain yield by 2–3% Grain ripening: Does not increase grain yield	[117,121]
Transgenic maize with homologous *ZmNF-YB2*	Increased grain yield by 50%	[75]
Transgenic maize preserves RNA stability and translation of cold shock protein B	Maintain cell function under water stress conditions	[122]
Gene knockout using CRISPR/Cas9	Generating *abh2*-CRISPR knockout drought-tolerant maize: *i1, d2*, and *d35*	[120]
Gene knockout using CRISPR/Cas9 Delivery: *Agrobacterium*	Generating *ZmSRL5*-CRISPR knockout maize: KO1, KO2 *ZmSRL5* is involved in drought tolerance by maintaining waxy structures in maize	[118]
CRISPR/Cas9 Delivery: *Agrobacterium*	Generating *zmslac1-2* and *zmcpk37* mutant-maize ZmSLAC1: stomatal closure in maize ZmCPK35 and ZmCPK37: improve drought tolerance and reduce yield loss under drought stress	[119]

Abbreviations: CRISPR/Cas9 Clustered Regularly Interspaced Short Palindromic Repeats/CRISPR-associated protein9.

**Table 4:** Factors for consideration in maize breeding in response to climate change.

**Factors**	**Description**	**References**
High-temperature tolerance	Development of maize varieties resistant to high temperatures; Selection and improvement of maize varieties capable of maintaining growth and productivity.	[125,126,127,128,129,130,131]
Drought tolerance	Efficient management and water use in maize variety development; Maintain survival and productivity in drought conditions.	[132,133,134,135]
Response to low precipitation	Developing maize varieties for water use efficiency	[136,137,138,139,140,141]

## Data Availability

Not applicable.

## References

[1] Lee H., Calvin K., Dasgupta D., Krinner G., Mukherji A., Thorne P., Trisos C., Romero J., Aldunce P., Barrett K. (2023). AR6 Synthesis Report: Climate Change 2023. Summary for Policymakers. https://www.ipcc.ch/report/ar6/syr/downloads/report/IPCC_AR6_SYR_SPM.pdf.

[2] Aliniaeifard S., Rezayian M., Mousavi S.H. (2023). Drought stress: Involvement of Plant Hormones in Perception, Signaling, and response. Plant Hormones and Climate Change.

[3] Khalili M., Naghavi M.R., Aboughadareh A.P., Rad H.N. (2013). Effects of drought stress on yield and yield components in maize cultivars (*Zea mays* L.). Int. J. Agron. Plant Prod..

[4] Song K., Kim K., Kim H., Moon J., Kim J., Baek S., Kwon Y., Lee B. (2015). Evaluation of drought tolerance in maize seedling using leaf rolling. Korean J. Crop Sci. Jakmul Hakhoe Chi.

[5] Lu Y., Hao Z., Xie C., Crossa J., Araus J.-L., Gao S., Vivek B.S., Magorokosho C., Mugo S., Makumbi D. (2011). Large-scale screening for maize drought resistance using multiple selection criteria evaluated under water-stressed and well-watered environments. Field Crop. Res..

[6] Saglam A., Kadioglu A., Demiralay M., Terzi R. (2014). Leaf rolling reduces photosynthetic loss in maize under severe drought. Acta Bot. Croat..

[7] Effendi R., Priyanto S.B., Aqil M., Azrai M. (2019). Drought adaptation level of maize genotypes based on leaf rolling, temperature, relative moisture content, and grain yield parameters. IOP Conf. Ser. Earth Environ. Sci..

[8] Aslam M., Zamir M.S.I., Afzal I., Yaseen M., Mubeen M., Shoaib A. (2013). Drought stress, its effect on maize production and development of drought tolerance through potassium application. Agron. Res. Mold..

[9] Sah R.P., Chakraborty M., Prasad K., Pandit M., Tudu V.K., Chakravarty M.K., Narayan S.C., Rana M., Moharana D. (2020). Impact of water deficit stress in maize: Phenology and yield components. Sci. Rep..

[10] Herrero M.P., Johnson R.R. (1981). Drought stress and its effects on maize reproductive systems. Crop Sci..

[11] Roy S.K., Cho S.W., Kwon S.J., Kamal A.H.M., Kim S.W., Oh M.W., Lee M.S., Chung K.Y., Xin Z., Woo S.H. (2016). Morpho-physiological and proteome level responses to cadmium stress in sorghum. PLoS ONE.

[12] Siebers N., Godlinski F., Leinweber P. (2014). Bone char as phosphorus fertilizer involved in cadmium immobilization in lettuce, wheat, and potato cropping. J. Plant Nutr. Soil Sci..

[13] Retamal-Salgado J., Hirzel J., Walter I., Matus I. (2017). Bioabsorption and bioaccumulation of cadmium in the straw and grain of maize (*Zea mays* L.) in growing soils contaminated with cadmium in different environment. Int. J. Environ. Res. Public Health.

[14] Marquez J.E., Pourret O., Faucon M.P., Weber S., Hoàng T.B.H., Martinez R.E. (2018). Effect of Cadmium, Copper and lead on the growth of rice in the coal mining region of Quang Ninh, Cam-Pha (Vietnam). Sustainability.

[15] Naveed M., Mustafa A., Majeed S., Naseem Z., Saeed Q. (2020). Through *Enterobacter* sp. MN17 Inoculation Together. Plants.

[16] Javed N., Ashraf M., Akram N.A., Al-Qurainy F. (2011). Alleviation of adverse effects of drought stress on growth and some potential physiological attributes in maize (*Zea mays* L.) by seed electromagnetic treatment. Photochem. Photobiol..

[17] Van Slycken S., Witters N., Meers E., Peene A., Michels E., Adriaensen K., Ruttens A., Vangronsveld J., Du Laing G., Wierinck I. (2013). Safe use of metal-contaminated agricultural land by cultivation of energy maize (*Zea mays*). Environ. Pollut..

[18] Huang R., Dong M., Mao P., Zhuang P., Paz-Ferreiro J., Li Y., Li Y., Hu X., Netherway P., Li Z. (2020). Evaluation of phytoremediation potential of five Cd (hyper)accumulators in two Cd contaminated soils. Sci. Total Environ..

[19] Raza A., Habib M., Kakavand S.N., Zahid Z., Zahra N., Sharif R., Hasanuzzaman M. (2020). Phytoremediation of cadmium: Physiological, biochemical, and molecular mechanisms. Biology.

[20] Fahad S., Bajwa A.A., Nazir U., Anjum S.A., Farooq A., Zohaib A., Sadia S., Nasim W., Adkins S., Saud S. (2017). Crop production under drought and heat stress: Plant responses and management options. Front. Plant Sci..

[21] Daryanto S., Wang L., Jacinthe P.-A. (2016). Global synthesis of drought effects on maize and wheat production. PLoS ONE.

[22] Lauer J. (2006). Concerns about Drought as Corn Pollination Begins. Field Crop..

[23] Nielsen R.L. (2002). Drought and heat stress effects on corn pollination. J. Agron..

[24] Nielsen R.L. (2002). A Fast & Accurate Pregnancy Test for Corn. http://www.kingcorn.org/news/timeless/EarShake.html.

[25] Sánchez B., Rasmussen A., Porter J.R. (2014). Temperatures and the growth and development of maize and rice: A review. Glob. Chang. Biol..

[26] Calleja-Cabrera J., Boter M., Oñate-Sánchez L., Pernas M. (2020). Root growth adaptation to climate change in crops. Front. Plant Sci..

[27] Jones C.A., Kiniry J.R., Dyke P.T. (1986). CERES-Maize: A Simulation Model of Maize Growth and Development.

[28] Lobell D.B., Burke M.B. (2010). On the use of statistical models to predict crop yield responses to climate change. Agric. For. Meteorol..

[29] Stone P. (2023). The effects of heat stress on cereal yield and quality. Crop Responses and Adaptations to Temperature Stress.

[30] Schoper J.B., Lambert R.J., Vasilas B.L. (1987). Pollen viability, pollen shedding, and combining ability for tassel heat tolerance in maize. Crop Sci..

[31] Sinsawat V., Leipner J., Stamp P., Fracheboud Y. (2004). Effect of heat stress on the photosynthetic apparatus in maize (*Zea mays* L.) grown at control or high temperature. Environ. Exp. Bot..

[32] Hussain T., Khan I.A., Malik M.A., Ali Z. (2006). Breeding potential for high temperature tolerance in corn (*Zea mays* L.). Pakistan J. Bot..

[33] McNellie J.P., Chen J., Li X., Yu J. (2018). Genetic mapping of foliar and tassel heat stress tolerance in maize. Crop Sci..

[34] Shao R., Yu K., Li H., Jia S., Yang Q., Zhao X., Zhao Y., Liu T. (2021). The effect of elevating temperature on the growth and development of reproductive organs and yield of summer maize. J. Integr. Agric..

[35] Liu P., Yin B., Gu L., Zhang S., Ren J., Wang Y., Duan W., Zhen W. (2023). Heat stress affects tassel development and reduces the kernel number of summer maize. Front. Plant Sci..

[36] Claassen M.M., Shaw R.H. (1970). Water deficit effects on corn. II. Grain components. Agron. J..

[37] Westgate M.E., Grant D.L.T. (1989). Water deficits and reproduction in maize: Response of the reproductive tissue to water deficits at anthesis and mid-grain fill. Plant Physiol..

[38] NeSmith D.S., Ritchie J.T. (1992). Effects of soil water-deficits during tassel emergence on development and yield component of maize (*Zea mays*). Field Crop. Res..

[39] Udomprasert N., Kijjanon J., Chusri-Iam K., Machuay A. (2005). Effects of Water Deficit at Tasseling on Photosynthesis, Development, and Yield of Corn. Kasetsart J. (Nat. Sci.).

[40] Heinigre R.W. (2000). Irrigation and Drought Management. https://www.scirp.org/%28S%28351jmbntvnsjt1aadkozje%29%29/reference/referencespapers.aspx?referenceid=1315258.

[41] Nam H.-H., Seo M.-C., Cho H.-S., Lee Y.-H., Seo Y.-J. (2017). Growth and yield responses of corn (*Zea mays* L.) as affected by growth period and irrigation intensity. Korean J. Soil Sci. Fertil..

[42] Gupta A., Rico-Medina A., Caño-Delgado A.I. (2020). The physiology of plant responses to drought. Science.

[43] Jaldhani V., Rao D.S., Beulah P., Nagaraju P., Suneetha K., Veronica N., Kondamudi R., Sundaram R.M., Madhav M.S., Neeraja C.N. (2022). Drought and heat stress combination in a changing climate. Climate Change and Crop Stress.

[44] Ray D.K., Gerber J.S., MacDonald G.K., West P.C. (2015). Climate variation explains a third of global crop yield variability. Nat. Commun..

[45] Shaw R.H. (1988). Climate requirement. Corn Corn Improv..

[46] Legg S. (2021). IPCC, 2021: Climate change 2021-the physical science basis. Interaction.

[47] Wahid A., Gelani S., Ashraf M., Foolad M.R. (2007). Heat tolerance in plants: An overview. Environ. Exp. Bot..

[48] Naveenkumar K.L., Sen D., Khanna V.K. (2018). Effect of maize production in a changing climate: Its impacts, adaptation, and mitigation strategies through breeding. Open Access J. Oncol. Med..

[49] Duveiller E., Singh R.P., Nicol J.M. (2007). The challenges of maintaining wheat productivity: Pests, diseases, and potential epidemics. Euphytica.

[50] Schussler J.R., Westgate M.E. (1991). Maize kernel set at low water potential: I. Sensitivity to reduced assimilates during early kernel growth. Crop Sci..

[51] Abrecht D.G., Carberry P.S. (1993). The influence of water deficit prior to tassel initiation on maize growth, development and yield. Field Crop. Res..

[52] Bolaños J., Edmeades G.O. (1993). Eight cycles of selection for drought tolerance in lowland tropical maize. II. Responses in reproductive behavior. Field Crop. Res..

[53] Ribaut J.-M., Hoisington D.A., Deutsch J.A., Jiang C., Gonzalez-de-Leon D. (1996). Identification of quantitative trait loci under drought conditions in tropical maize. 1. Flowering parameters and the anthesis-silking interval. Theor. Appl. Genet..

[54] Serna L. (2022). Maize stomatal responses against the climate change. Front. Plant Sci..

[55] Maitah M., Malec K., Maitah K. (2021). Influence of precipitation and temperature on maize production in the Czech Republic from 2002 to 2019. Sci. Rep..

[56] Khan A., Ali S., Shah S.A., Khan A., Ullah R. (2019). Impact of climate change on maize productivity in Khyber Pakhtunkhwa, Pakistan. Sarhad J. Agric..

[57] Webber H., Ewert F., Olesen J.E., Müller C., Fronzek S., Ruane A.C., Bourgault M., Martre P., Ababaei B., Bindi M. (2018). Diverging importance of drought stress for maize and winter wheat in Europe. Nat. Commun..

[58] Dellal İ., McCarl B.A., Butt T. (2011). The economic assessment of climate change on Turkish agriculture. J. Environ. Prot. Ecol..

[59] Yu C., Miao R., Khanna M. (2021). Maladaptation of US corn and soybeans to a changing climate. Sci. Rep..

[60] Notununu I., Moleleki L., Roopnarain A., Adeleke R. (2022). Effects of plant growth-promoting rhizobacteria on the molecular responses of maize under drought and heat stresses: A review. Pedosphere.

[61] Pei Y.-Y., Lei L., Fan X.-W., Li Y.-Z. (2023). Effects of high air temperature, drought, and both combinations on maize: A case study. Plant Sci..

[62] Kooyers N.J. (2015). The evolution of drought escape and avoidance in natural herbaceous populations. Plant Sci..

[63] Shavrukov Y., Kurishbayev A., Jatayev S., Shvidchenko V., Zotova L., Koekemoer F., De Groot S., Soole K., Langridge P. (2017). Early flowering as a drought escape mechanism in plants: How can it aid wheat production?. Front. Plant Sci..

[64] Cattivelli L., Rizza F., Badeck F.-W., Mazzucotelli E., Mastrangelo A.M., Francia E., Marè C., Tondelli A., Stanca A.M. (2008). Drought tolerance improvement in crop plants: An integrated view from breeding to genomics. Field Crop. Res..

[65] Tardieu F., Simonneau T., Muller B. (2018). The physiological basis of drought tolerance in crop plants: A scenario-dependent probabilistic approach. Annu. Rev. Plant Biol..

[66] Saini H.S., Westgate M.E. (1999). Reproductive development in grain crops during drought. Adv. Agron..

[67] Boyer J.S., Westgate M.E. (2004). Grain yields with limited water. J. Exp. Bot..

[68] Lobell D.B., Roberts M.J., Schlenker W., Braun N., Little B.B., Rejesus R.M., Hammer G.L. (2014). Greater sensitivity to drought accompanies maize yield increase in the US Midwest. Science.

[69] Babu R.C., Nguyen B.D., Chamarerk V., Shanmugasundaram P., Chezhian P., Jeyaprakash P., Ganesh S.K., Palchamy A., Sadasivam S., Sarkarung S. (2003). Genetic analysis of drought resistance in rice by molecular markers: Association between secondary traits and field performance. Crop Sci..

[70] Tester M., Langridge P. (2010). Breeding technologies to increase crop production in a changing world. Science.

[71] Liu S., Qin F. (2021). Genetic dissection of maize drought tolerance for trait improvement. Mol. Breed..

[72] Voytas D.F., Gao C. (2014). Precision genome engineering and agriculture: Opportunities and regulatory challenges. PLoS Biol..

[73] Gao C. (2015). Genome editing in crops: From bench to field. Natl. Sci. Rev..

[74] Yang S., Vanderbeld B., Wan J., Huang Y. (2010). Narrowing down the targets: Towards successful genetic engineering of drought-tolerant crops. Mol. Plant.

[75] Nelson D.E., Repetti P.P., Adams T.R., Creelman R.A., Wu J., Warner D.C., Anstrom D.C., Bensen R.J., Castiglioni P.P., Donnarummo M.G. (2007). Plant nuclear factor Y (NF-Y) B subunits confer drought tolerance and lead to improved corn yields on water-limited acres. Proc. Natl. Acad. Sci. USA.

[76] Zeng T., Zhang D., Li Y., Li C., Liu X., Shi Y., Song Y., Li Y., Wang T. (2020). Identification of genomic insertion and flanking sequences of the transgenic drought-tolerant maize line “SbSNAC1-382” using the single-molecule real-time (SMRT) sequencing method. PLoS ONE.

[77] Wang B., Li Z., Ran Q., Li P., Peng Z., Zhang J. (2018). ZmNF-YB16 overexpression improves drought resistance and yield by enhancing photosynthesis and the antioxidant capacity of maize plants. Front. Plant Sci..

[78] Muppala S., Gudlavalleti P.K., Malireddy K.R., Puligundla S.K., Dasari P. (2021). Development of stable transgenic maize plants tolerant for drought by manipulating ABA signaling through Agrobacterium-mediated transformation. J. Genet. Eng. Biotechnol..

[79] Quan R., Shang M., Zhang H., Zhao Y., Zhang J. (2004). Engineering of enhanced glycine betaine synthesis improves drought tolerance in maize. Plant Biotechnol. J..

[80] Liu Y., Qin L., Han L., Xiang Y., Zhao D. (2015). Overexpression of maize SDD1 (ZmSDD1) improves drought resistance in Zea mays L. by reducing stomatal density. Plant Cell Tissue Organ Cult..

[81] Wang X., Wang H., Liu S., Ferjani A., Li J., Yan J., Yang X., Qin F. (2016). Genetic variation in ZmVPP1 contributes to drought tolerance in maize seedlings. Nat. Genet..

[82] Virlouvet L., Jacquemot M.-P., Gerentes D., Corti H., Bouton S., Gilard F., Valot B., Trouverie J., Tcherkez G., Falque M. (2011). The ZmASR1 protein influences branched-chain amino acid biosynthesis and maintains kernel yield in maize under water-limited conditions. Plant Physiol..

[83] Xu Z., Wang M., Guo Z., Zhu X., Xia Z. (2019). Identification of a 119-bp promoter of the maize sulfite oxidase gene (ZmSO) that confers high-level gene expression and ABA or drought inducibility in transgenic plants. Int. J. Mol. Sci..

[84] Chen Z., Liu Y., Yin Y., Liu Q., Li N., Li X., He W., Hao D., Liu X., Guo C. (2019). Expression of AtGA2ox1 enhances drought tolerance in maize. Plant Growth Regul..

[85] Sheoran S., Gupta M., Kumari S., Kumar S., Rakshit S. (2022). Meta-QTL analysis and candidate genes identification for various abiotic stresses in maize (*Zea mays* L.) and their implications in breeding programs. Mol. Breed..

[86] Welcker C., Boussuge B., Bencivenni C., Ribaut J.M., Tardieu F. (2007). Are source and sink strengths genetically linked in maize plants subjected to water deficit? A QTL study of the responses of leaf growth and of anthesis-silking interval to water deficit. J. Exp. Bot..

[87] Ruta N., Liedgens M., Fracheboud Y., Stamp P., Hund A. (2010). QTLs for the elongation of axile and lateral roots of maize in response to low water potential. Theor. Appl. Genet..

[88] Liu Y., Subhash C., Yan J., Song C., Zhao J., Li J. (2011). Maize leaf temperature responses to drought: Thermal imaging and quantitative trait loci (QTL) mapping. Environ. Exp. Bot..

[89] Landi P., Giuliani S., Salvi S., Ferri M., Tuberosa R., Sanguineti M.C. (2010). Characterization of root-yield-1.06, a major constitutive QTL for root and agronomic traits in maize across water regimes. J. Exp. Bot..

[90] Hao Z.F., Li X.H., Xie C.X., Li M.S., Zhang D.G., Bai L., Zhang S.H. (2008). Two consensus quantitative trait loci clusters controlling anthesis–silking interval, ear setting and grain yield might be related with drought tolerance in maize. Ann. Appl. Biol..

[91] Agrama H.A.S., Moussa M.E. (1996). Mapping QTLs in breeding for drought tolerance in maize (*Zea mays* L.). Euphytica.

[92] Almeida G.D., Makumbi D., Magorokosho C., Nair S., Borém A., Ribaut J.-M., Bänziger M., Prasanna B.M., Crossa J., Babu R. (2013). QTL mapping in three tropical maize populations reveals a set of constitutive and adaptive genomic regions for drought tolerance. Theor. Appl. Genet..

[93] Tuberosa R., Sanguineti M.C., Landi P., Salvi S., Casarini E., Conti S. (1998). RFLP mapping of quantitative trait loci controlling abscisic acid concentration in leaves of drought-stressed maize (*Zea mays* L.). Theor. Appl. Genet..

[94] Sanguineti M.C., Tuberosa R., Landi P., Salvi S., Maccaferri M., Casarini E., Conti S. (1999). QTL analysis of drought-related traits and grain yield in relation to genetic variation for leaf abscisic acid concentration in field-grown maize. J. Exp. Bot..

[95] Abdelghany M., Liu X., Hao L., Gao C., Kou S., Su E., Zhou Y., Wang R., Zhang D., Li Y. (2019). QTL analysis for yield-related traits under different water regimes in maize. Maydica.

[96] Leng P., Khan S.U., Zhang D., Zhou G., Zhang X., Zheng Y., Wang T., Zhao J. (2022). Linkage mapping reveals QTL for flowering time-related traits under multiple abiotic stress conditions in maize. Int. J. Mol. Sci..

[97] Hu X., Wang G., Du X., Zhang H., Xu Z., Wang J., Chen G., Wang B., Li X., Chen X. (2021). QTL analysis across multiple environments reveals promising chromosome regions associated with yield-related traits in maize under drought conditions. Crop J..

[98] Semagn K., Beyene Y., Warburton M.L., Tarekegne A., Mugo S., Meisel B., Sehabiague P., Prasanna B.M. (2013). Meta-analyses of QTL for grain yield and anthesis silking interval in 18 maize populations evaluated under water-stressed and well-watered environments. BMC Genom..

[99] Zhao X.Q., Zhong Y. (2021). Genetic dissection of the photosynthetic parameters of maize (*Zea mays* L.) in drought-stressed and well-watered environments. Russ. J. Plant Physiol..

[100] Zhang F., Zhang J., Ma Z., Xia L., Wang X., Zhang L., Ding Y., Qi J., Mu X., Zhao F. (2021). Bulk analysis by resequencing and RNA-seq identifies candidate genes for maintaining leaf water content under water deficit in maize. Physiol. Plant..

[101] Araus J.L., Slafer G.A., Royo C., Serret M.D. (2008). Breeding for yield potential and stress adaptation in cereals. CRC Crit. Rev. Plant Sci..

[102] Collins N.C., Tardieu F., Tuberosa R. (2008). Quantitative trait loci and crop performance under abiotic stress: Where do we stand?. Plant Physiol..

[103] Eathington S.R., Crosbie T.M., Edwards M.D., Reiter R.S., Bull J.K. (2007). Molecular markers in a commercial breeding program. Crop Sci..

[104] Tsonev S., Todorovska E., Avramova V., Kolev S., Abu-Mhadi N., Christov N.K. (2009). Genomics assisted improvement of drought tolerance in maize: QTL approaches. Biotechnol. Biotechnol. Equip..

[105] Xu Y., Skinner D.J., Wu H., Palacios-Rojas N., Araus J.L., Yan J., Gao S., Warburton M.L., Crouch J.H. (2009). Advances in maize genomics and their value for enhancing genetic gains from breeding. Int. J. Plant Genom..

[106] Stuber C.W., Edwards M.D., Wendel J.F. (1987). Molecular Marker-Facilitated Investigations of Quantitative Trait Loci in Maize. II. Factors Influencing Yield and its Component Traits. Crop Sci..

[107] Lebreton C., Lazić-Jančić V., Steed A., Pekić S., Quarrie S.A. (1995). Identification of QTL for drought responses in maize and their use in testing causal relationships between traits. J. Exp. Bot..

[108] Messmer R., Fracheboud Y., Bänziger M., Vargas M., Stamp P., Ribaut J.M. (2009). Drought stress and tropical maize: QTL-by-environment interactions and stability of QTLs across environments for yield components and secondary traits. Theor. Appl. Genet..

[109] Szalma S.J., Hostert B.M., LeDeaux J.R., Stuber C.W., Holland J.B. (2007). QTL mapping with near-isogenic lines in maize. Theor. Appl. Genet..

[110] Tuberosa R., Salvi S., Sanguineti M.C., Landi P., Maccaferri M., Conti S. (2002). Mapping QTLS regulating morpho-physiological traits and yield: Case studies, shortcomings and perspectives in drought-stressed maize. Ann. Bot..

[111] Campos H., Cooper M., Habben J.E., Edmeades G.O., Schussler J.R. (2004). Improving drought tolerance in maize: A view from industry. Field Crop. Res..

[112] Thatcher S.R., Danilevskaya O.N., Meng X., Beatty M., Zastrow-Hayes G., Harris C., Van Allen B., Habben J., Li B. (2016). Genome-wide analysis of alternative splicing during development and drought stress in maize. Plant Physiol..

[113] Danilevskaya O.N., Yu G., Meng X., Xu J., Stephenson E., Estrada S., Chilakamarri S., Zastrow-Hayes G., Thatcher S. (2019). Developmental and transcriptional responses of maize to drought stress under field conditions. Plant Direct.

[114] Lin M., Qiao P., Matschi S., Vasquez M., Ramstein G.P., Bourgault R., Mohammadi M., Scanlon M.J., Molina I., Smith L.G. (2022). Integrating GWAS and TWAS to elucidate the genetic architecture of maize leaf cuticular conductance. Plant Physiol..

[115] Zhu Z., Zhang F., Hu H., Bakshi A., Robinson M.R., Powell J.E., Montgomery G.W., Goddard M.E., Wray N.R., Visscher P.M. (2016). Integration of summary data from GWAS and eQTL studies predicts complex trait gene targets. Nat. Genet..

[116] Wang T., Zhang H., Zhu H. (2019). CRISPR technology is revolutionizing the improvement of tomato and other fruit crops. Hortic. Res..

[117] Shi J., Gao H., Wang H., Lafitte H.R., Archibald R.L., Yang M., Hakimi S.M., Mo H., Habben J.E. (2017). ARGOS 8 variants generated by CRISPR-Cas9 improve maize grain yield under field drought stress conditions. Plant Biotechnol. J..

[118] Pan Z., Liu M., Zhao H., Tan Z., Liang K., Sun Q., Gong D., He H., Zhou W., Qiu F. (2020). ZmSRL5 is involved in drought tolerance by maintaining cuticular wax structure in maize. J. Integr. Plant Biol..

[119] Li X., Gao Y., Wu W., Chen L., Wang Y. (2022). Two calcium-dependent protein kinases enhance maize drought tolerance by activating anion channel ZmSLAC1 in guard cells. Plant Biotechnol. J..

[120] Liu S., Li C., Wang H., Wang S., Yang S., Liu X., Yan J., Li B., Beatty M., Zastrow-Hayes G. (2020). Mapping regulatory variants controlling gene expression in drought response and tolerance in maize. Genome Biol..

[121] Shi J., Habben J.E., Archibald R.L., Drummond B.J., Chamberlin M.A., Williams R.W., Lafitte H.R., Weers B.P. (2015). Overexpression of ARGOS genes modifies plant sensitivity to ethylene, leading to improved drought tolerance in both Arabidopsis and maize. Plant Physiol..

[122] Sammons B., Whitsel J., Stork L.G., Reeves W., Horak M. (2014). Characterization of drought-tolerant maize MON 87460 for use in environmental risk assessment. Crop Sci..

[123] Tesfaye K., Kruseman G., Cairns J.E., Zaman-Allah M., Wegary D., Zaidi P.H., Boote K.J., Rahut D., Erenstein O. (2018). Potential benefits of drought and heat tolerance for adapting maize to climate change in tropical environments. Clim. Risk Manag..

[124] Jiang L.-G., Li B., Liu S.-X., Wang H.-W., Li C.-P., Song S.-H., Beatty M., Zastrow-Hayes G., Yang X.-H., Qin F. (2019). Characterization of Proteome Variation During Modern Maize Breeding. Mol. Cell. Proteom..

[125] EL Sabagh A., Hossain A., Aamir Iqbal M., Barutçular C., Islam M.S., Çiğ F., Erman M., Sytar O., Brestic M., Wasaya A. (2021). Maize Adaptability to Heat Stress under Changing Climate. Plant Stress Physiology.

[126] Chukwudi U.P., Kutu F.R., Mavengahama S. (2021). Influence of heat stress, variations in soil type, and soil amendment on the growth of three drought–tolerant maize varieties. Agronomy.

[127] Niu S., Du X., Wei D., Liu S., Tang Q., Bian D., Zhang Y., Cui Y., Gao Z. (2021). Heat stress after pollination reduces kernel number in maize by insufficient assimilates. Front. Genet..

[128] Li Y., Han X., Ren H., Zhao B., Zhang J., Ren B., Gao H., Liu P. (2023). Exogenous SA or 6-BA maintains photosynthetic activity in maize leaves under high temperature stress. Crop J..

[129] Ahmad M., Imtiaz M., Nawaz S., Mubeen F., Sarwar Y., Hayat M., Asif M., Naqvi R., Imran A. (2023). Thermotolerant PGPR consortium B3P modulates physio-biochemical and molecular machinery for enhanced heat tolerance in maize during early vegetative growth. Ann. Microbiol..

[130] Chekole F.C., Mohammed Ahmed A. (2023). Future climate implication on maize (*Zea mays*) productivity with adaptive options at Harbu district, Ethiopia. J. Agric. Food Res..

[131] Ghani A., Yousaf M.I., Hussain K., Hussain S., Razaq A., Akhtar N., Ibrar I., Kamal N., Ali B., Khan A.M. (2023). Relationship between high-temperature stress and key physio-chemical, reactive oxygen species and antioxidants in spring maize hybrids under semi-arid conditions. Biol. Clin. Sci. Res. J..

[132] Muruo R.M., Nchore S.B., Oduor R.O., Ngugi M.P. (2023). Overexpressing the IPT gene improves drought tolerance and nutritional value of tropical maize (*Zea mays* L.). bioRxiv.

[133] Mansour E., El-Sobky E.S.E.A., Abdul-Hamid M.I.E., Abdallah E., Zedan A.M.I., Serag A.M., Silvar C., El-Hendawy S., Desoky E.S.M. (2023). Enhancing Drought Tolerance and Water Productivity of Diverse Maize Hybrids (Zea mays) Using Exogenously Applied Biostimulants under Varying Irrigation Levels. Agronomy.

[134] Messina C.D., Gho C., Hammer G.L., Cooper M. (2023). Two decades of harnessing standing genetic variation for physiological traits to improve drought tolerance in maize (*Zea mays* L.). J. Exp. Bot..

[135] Nihranz C.T., Guzchenko I.A., Casteel C.L. (2023). Silencing ZmPP2C-A10 with a foxtail mosaic virus (FoMV) derived vector benefits maize growth and development following water limitation. Plant Biol..

[136] Abaza A.S.D., Elshamly A.M.S., Alwahibi M.S., Elshikh M.S., Ditta A. (2023). Impact of different sowing dates and irrigation levels on NPK absorption, yield and water use efficiency of maize. Sci. Rep..

[137] Xu J., Mu Q., Ding Y., Sun S., Zou Y., Yu L., Zhang P., Yang N., Guo W., Cai H. (2023). Considering spatio-temporal dynamics of soil water with evapotranspiration partitioning helps to clarify water utilization characteristics of summer maize under deficit irrigation. J. Hydrol..

[138] Te X., Din A.M.U., Cui K., Raza M.A., Faraz Ali M., Xiao J., Yang W. (2023). Inter-specific root interactions and water use efficiency of maize/soybean relay strip intercropping. Field Crop. Res..

[139] Setti A., Castelli G., Villani L., Ferrise R., Bresci E. (2023). Modelling the impacts of water harvesting and climate change on rainfed maize yields in Senegal. J. Agric. Eng..

[140] Jiao F., Hong S., Zhang Q., Li M., Shi R., Ma Y., Li Q. (2023). Subsoiling before winter wheat cultivation increases photosynthetic characteristics and leaf water-use efficiency of summer maize in a double-cropping system. Arch. Agron. Soil Sci..

[141] Hütsch B.W., Schubert S. (2023). Grain yield, harvest index, water-use efficiency and nitrogen partitioning to grain can be improved by application of the plant growth regulator paclobutrazol to maize plants with reduced N supply. J. Agron. Crop Sci..

